# Interleukin-1 inhibition, chronic kidney disease-mineral and bone disorder, and physical function 

**DOI:** 10.5414/CN109122

**Published:** 2017-07-12

**Authors:** Kristen L. Nowak, Adriana Hung, Talat Alp Ikizler, Heather Farmer-Bailey, Natjalie Salas-Cruz, Sudipa Sarkar, Andrew Hoofnagle, Zhiying You, Michel Chonchol

**Affiliations:** 1 University of Colorado Denver Anschutz Medical Campus, Aurora, CO,; 2 Vanderbilt University Medical Center,; 3 VA Tennessee Valley Healthcare System,; 4 Vanderbilt Center for Kidney Disease, Nashville, TN, and; 5 University of Washington, Seattle, WA, USA

**Keywords:** inflammation, clinical trial, fibroblast growth factor 23, mineral metabolism, vitamin D

## Abstract

Objective: Epidemiologic studies have suggested a link between chronic systemic inflammation and chronic kidney disease-mineral and bone disorder (CKD-MBD). Additionally, declining renal function is associated with worsening physical and cognitive function, which may potentially be explained by systemic inflammation, CKD-MBD, or both. We hypothesized that inhibiting inflammation with an interleukin-1 (IL-1) trap would improve markers of CKD-MBD as well as physical/cognitive function in patients with moderate-to-severe CKD. Methods: In a two-site, double-blind trial, 39 patients with stage 3 – 4 CKD completed a randomized trial receiving either the IL-1 trap rilonacept (160 mg/week) or placebo for 12 weeks. The following CKD-MBD markers were assessed in serum before and after the intervention: calcium, phosphorus, 25-hydroxyvitamin D, 1,25-dihydroxyvitamin D, 24,25-dihydroxyvitamin D, intact parathyroid hormone (iPTH), and fibroblast growth factor 23 (FGF23). A battery of tests was also administered in a subgroup (n = 23) to assess multiple domains of physical function (endurance, locomotion, dexterity, balance, strength, and fatigue) and cognitive function. Results: Participants were 65 ± 10 years of age, 23% female, and had a mean estimated glomerular filtration rate of 38 ± 13 mL/min/1.73m^2^. There were no changes in serum calcium, phosphorus, any vitamin D metabolite, iPTH, or FGF23 levels (p ≥ 0.28) with IL-1 inhibition. Similarly, rilonacept did not alter locomotion, dexterity, balance, strength, fatigue, or cognitive function (p ≥ 0.13). However, endurance (400-m walk time) tended to improve in the rilonacept (–31 s) vs. placebo group (–2 s; p = 0.07). Conclusions: In conclusion, 12 weeks of IL-1 inhibition did not improve markers of CKD-MBD or physical function.

## Introduction 

Chronic kidney disease-mineral and bone disorder (CKD-MBD) is a common complication of CKD, occurring early in the disease and persisting across all stages [1]. It is characterized by progressive disturbances in serum concentrations of calcium, phosphorus, vitamin D metabolites, intact parathyroid hormone (iPTH), and fibroblast growth factor 23 (FGF23). Chronic inflammation, driven in part by high circulating levels of interleukin-1 (IL-1) and its naturally-occurring receptor antagonist [2, 3], is an additional distinguishing feature of CKD. 

Numerous epidemiologic studies have demonstrated an independent association between chronic inflammation and disturbed markers of mineral metabolism [4, 5, 6, 7]. Additionally, in vitro and in vivo studies suggest direct regulation of both FGF23 [8, 9, 10, 11] and 1,25-dihydroxyvitamin D (1,25(OH)_2_D) [12] production by inflammation. However, whether directly inhibiting inflammation in humans improves CKD-MBD abnormalities is unknown. 

Even in early stages of CKD, declining renal function is independently associated with worsening physical function across various domains [13, 14], which may potentially be explained by systemic inflammation, CKD-MBD, or both. Elevated levels of inflammatory markers (i.e., C-reactive protein (CRP), interleukin-6, IL-1 receptor antagonist) are associated with reduced physical performance [15, 16] as well as cognitive decline in older adults [17, 18]. Furthermore, IL-1 inhibition improves physical function in patients with rheumatoid arthritis, another disease process characterized by chronic inflammation [19, 20]. Thus, IL-1 inhibition may improve physical and/or cognitive function in patients with CKD. 

We recently completed a randomized, placebo-controlled trial that demonstrated that IL-1 inhibition is efficacious for improving vascular endothelial function as well as reducing systemic inflammation (high-sensitivity CRP (hsCRP)) in patients with stage 3 – 4 CKD [21]. As a secondary analysis in this trial, we evaluated changes in circulating markers of CKD-MBD in response to IL-1 inhibition as compared to placebo. Additionally, as a pilot study, we evaluated changes in physical as well as cognitive function in a subgroup of participants from the parent study. We present here the first direct evidence in humans regarding the effect of inhibiting IL-1 upon markers of mineral metabolism, as well as on physical and cognitive function. 

## Materials and methods 

### Study design 

Additional details of the study design and other methods are provided in the online-only data supplement. The details of the parent study and primary outcomes have been published previously [21]. Briefly, a 12-week, randomized, placebo-controlled (1 : 1 allocation), parallel-group, double-blind study was conducted at two sites (the University of Colorado Denver Anschutz Medical Campus and the Tennessee Valley Healthcare System/Vanderbilt University Medical Center) between September 2012 and September 2014. Secondary outcomes, determined a priori, that were evaluated in the present analysis were changes in serum markers of CKD-MBD and physical/cognitive function. 

### Study participants 

Detailed inclusion and exclusion criteria have been published previously and are described in the supplement [21]. All participants who completed the parent study were included in the analysis of markers of CKD-MBD (n = 19 rilonacept and n = 20 placebo). Three participants in the rilonacept group and 2 participants in the placebo group did not complete the parent study. A subgroup of participants from the Denver site additionally completed a battery of tests to evaluate various domains of cognitive and physical function (n = 12 rilonacept and n = 11 placebo). All procedures were approved by the Institutional Review Board of the University of Colorado Denver, the Tennessee Valley Healthcare System, and Vanderbilt University Medical Center. The nature, benefits, and risks of the study were explained to the volunteers, and their written informed consent was obtained prior to participation. 

### Procedures 

**Rilonacept and weekly visits**

These details have been published previously [21]. Briefly, rilonacept, a soluble IL-1 decoy receptor, or placebo was injected subcutaneously (320-mg loading dose followed by 160 mg/week) for 12 weeks. 

**Markers of CKD-MBD**

Serum samples were stored at –80^◦^C until time of analysis at the University of Washington (2015). Serum concentrations of 25-hydroxyvitamin D (25(OH)D, 1,25(OH)_2_D), 24,25-dihydroxyvitamin D (24,25(OH)_2_D_3_), intact FGF23, iPTH, calcium, and phosphorus were measured, as detailed in the supplement. 

**Physical and cognitive function**

These measurements were performed at the Health and Wellness Center at the University of Colorado Anschutz Medical Campus. Endurance, mobility, muscle strength, grip strength, balance, dexterity, cognitive function, and self-reported perception of fatigue were measured, as detailed in the supplement. 

### Statistics 

Differences in baseline variables between treatment groups were assessed using t-tests, rank-based tests, or χ^2^-tests. The changes in markers of CKD-MBD and physical/cognitive function in response to treatment were analyzed by comparing change from baseline for each outcome between study groups using a two-sample t-test. Skewed variables were log-transformed prior to analyses. No adjustment was made for multiple comparisons as all outcomes in this secondary analysis were considered exploratory. The evaluation of physical and cognitive function was performed as a pilot study in a subgroup of participants. All data are reported as mean ± SD or medians (interquartile range). SAS software (version 9.4) was used for all analyses. 

## Results 

### Enrollment and baseline clinical characteristics 

All participants who completed the parent study were included in the analysis of serum markers of CKD-MBD (n = 19 in the rilonacept group and n = 20 placebo in the placebo group). Similar to the parent study, which included all consented patients, participants in each arm (rilonacept and placebo) did not differ significantly in terms of baseline characteristics including: gender, race/ethnicity, etiology of CKD, medications, smoking status, eGFR, urine protein/creatinine ratio, body-mass index, blood pressure, and serum albumin (Table 1). A subgroup of participants from the Denver site additionally completed a battery of tests to evaluate various domains of physical and cognitive function (n = 12 rilonacept and n = 11 placebo). The subgroup did not differ significantly from the entire cohort in any baseline clinical characteristic (Supplemental Table 1). A comparison of baseline markers of CKD-MBD and measures of physical function according to study group is shown in Supplemental Table 2. Serum 24,25(OH)_2_D_3_ levels were higher in the placebo group compared to the rilonacept group at baseline (p < 0.01), but no other variables were significantly different between groups. 

### Effect of IL-1 inhibition on CKD-MBD 

Twelve weeks of IL-1 inhibition with rilonacept did not significantly change any of the measured markers of CKD-MBD (serum calcium, phosphorus, 25(OH)D, 1,25(OH)_2_D, 24,25(OH)_2_D_3_, intact parathyroid hormone (iPTH), and FGF23) as compared to the placebo group (Table 2). FGF-23 levels were not significantly reduced in both the rilonacept and placebo weeks after 12 weeks (p = 0.69 and p = 0.33, respectively). Of note, rilonacept significantly lowered hsCRP levels compared to placebo, as published previously [21]. 

### Effect of IL-1 inhibition on physical and cognitive function 

There were no significant changes in measures of physical function (endurance (400-m walk time), mobility (timed up and go test), muscle strength (chair stands), grip strength (hand dynamometer), dexterity (pegboard time), balance (rapid step test)), cognitive function (trail making tests A and B), or perceived fatigue (fatigue severity score) in the rilonacept compared to placebo group. However, there were some trends towards improvements, given that this was a hypothesis-generating pilot substudy with a small sample size. In particular, there was a nearly-significant trend towards reduced 400-m walk time with rilonacept as compared to placebo (Supplemental Figure 1)[Table Table3]. 

## Discussion 

In the first clinical trial to examine the effect of direct inhibition of IL-1 upon markers of CKD-MBD, we found no change in any circulating markers of mineral metabolism, including 25(OH)D, 1,25(OH)_2_D, and FGF23. These results are in contrast with epidemiologic evidence to date, which has suggested regulation of mineral metabolism by inflammation. A nonsignificant trend towards improved endurance, as measured by 400-m walk time, was observed in the rilonacept group in the substudy. 

In patients with CKD, an independent association has been demonstrated between elevated FGF23 and increased inflammatory markers, including IL-6, CRP, and tumor necrosis factor-α (TNF-α) [4, 5]. Both acute and chronic inflammation also stimulate FGF23 production in animal models [8]; however, animal studies have shown that FGF23 proteolytic cleavage is decreased to a greater extent than FGF23 transcription during chronic inflammation, ultimately leading to a slight increase in intact FGF23 [8]. In our study, in which we blocked important inflammatory pathways in a state of chronic inflammation, we did not observe any changes in intact FGF23. 

Similarly, an independent association between high serum phosphate or calcium × phosphate product and increased systemic inflammatory markers has been observed both in nondialysis-dependent CKD [6] and chronic dialysis patients [22]. However, the effect, of directly inhibiting inflammatory pathways in chronic inflammation, on markers of mineral metabolism has never been evaluated previously in humans. A couple of small studies have considered the opposite scenario, examining the effect of targeting CKD-MBD on markers of inflammation. In a single-arm study of 36 chronic hemodialysis patients, 24 weeks of treatment with sevelamer reduced serum phosphate and hsCRP, and the reduction in hsCRP correlated with the decrease in serum phosphate level [23]. Similarly, 1 year of cholecalciferol supplementation lowered CRP in chronic hemodialysis patients [24]. Thus, abnormalities of CKD-MBD appear to be proinflammatory; however, our results indicate that inhibiting chronic inflammation does not improve these abnormalities. 

There are several mechanisms that could potentially link markers of mineral metabolism and inflammation. High phosphate may trigger phosphorylation-driven inflammatory cascades [6, 25], possibly via klotho, a regulator of phosphate metabolism, through a nuclear factor κ B (NFκB)-dependent mechanism [26, 27]. Additionally, sevelamer has pleiotropic effects beyond phosphate lowering, including anti-inflammatory actions [28]. Recent in vitro and in vivo studies have suggested that chronic inflammation is a potent regulator in FGF23 production. Proinflammatory cytokines, including IL-1β, can stimulate FGF23 production in osteocytes [29], cardiac fibroblasts [30], and mouse models of CKD [8]. Inflammation may stimulate FGF23 production via NFκB signaling [10] or through a hypoxia-inducible factor 1-α (HIF-1α)-dependent mechanism [8]. High leptin may also mediate the association between FGF23 and inflammation as FGF23 is associated with high leptin [31], and leptin correlates with inflammation in patients with CKD [32]. Additionally, high-circulating FGF23 levels may reduce circulating levels of 1,25(OH)_2_D via inhibition of 1-α-hydroxylase [33]. 1,25(OH)_2_D inhibits TNF-α-induced NFκB activation [34] as well as the production of other proinflammatory cytokines [35]. In turn, inflammation may also downregulate the 1-α-hydroxylase promotor via NFκB [12]. However, contrary to these possibilities, our results indicate that directly inhibiting chronic inflammation does not improve circulating levels of 1,25(OH)_2_D or any of the other abnormalities of mineral metabolism common in patients with moderate-to-severe CKD. 

Even in early stages of CKD, there is evidence of worsening physical function [13, 14] and cognitive decline [36, 37], which are associated with adverse outcomes including increased rate of hospitalization, mortality, and reduced quality of life [38, 39]. Chronic inflammation may contribute to a decline in physical function in patients with CKD via muscle catabolism [40]. In addition, inflammation may mediate cognitive impairment via vascular dysfunction and cerebrovascular disease [41]. Thus, IL-1 inhibition may improve physical and/or cognitive function in patients with CKD. While this study was not powered to detect changes in physical and cognitive function in response to IL-1 inhibition with rilonacept, and these measurements were only performed in a subgroup as a pilot study, the results suggest that this hypothesis should be considered further in a larger population of CKD patients. In particular, there was a near-significant trend towards improved endurance, as measured by 400-m walk time, in the rilonacept compared to placebo group. However, these results should be interpreted cautiously given the nature of analyses of secondary outcomes and a small sample size, and it is possible the change in 400-m walk time may represent a regression to the mean. Additionally, as the study was only 12 weeks in duration, it is possible that a longer duration of IL-1 inhibition, or perhaps treating patients with an earlier stage of CKD, may indeed alter markers of CKD-MBD and/or indices of physical and cognitive function. Finally, we measured intact FGF23, and it is possible that additional assessment of C-terminal FGF23 fragments, which are presumed to be biologically inactive, may have yielded different results [42]. 

The major strength of this study is that it is the first randomized controlled trial to evaluate changes in serum markers of CKD-MBD in response to direct inhibition of IL-1. Additional novelty and hypothesis-generating data were provided by the assessment of physical and cognitive function in a subgroup of participants. The results are limited by the fact that these outcomes, while determined a priori, are secondary outcomes that the trial was not powered to evaluate, particularly for the pilot substudy of physical and cognitive function. Given the sample size required to power the primary outcomes in the parent study, the sample size in this analysis is also relatively small, limiting our potential not only to detect a small effect size, but also to perform multidimensional subgroup analysis that could allow for identification of specific subgroups of patients who may be more likely to have changes in markers of CKD-MBD (e.g., the highly inflamed, those with the lowest bicarbonate levels). 

In conclusion, direct inhibition of IL-1 does not improve serum markers of mineral metabolism in patients with moderate-to-severe-CKD. Future research with larger sample sizes should evaluate further whether IL-1 inhibition may improve physical or cognitive function in this population. 

## Conflict of interest 

Study drug and matching placebo were kindly provided by Regeneron Pharmaceuticals, Tarrytown, New York, NY, USA. A.H. receives grant support from Waters Inc. The sponsors had no influence on the design, execution, or analysis of the results of the study. 

## Acknowledgments 

This work was supported by the American Heart Association postdoctoral fellowship award 12POST11920023 for K.L.N., the US Department of Veterans Affairs CSR&D office Career Development Award 2-031-09S for A.M.H. Additional support was provided by the National Institutes of Health (NIH) National Center for Research Resources Colorado CTSI UL1 RR025780, NIH National Center for Advancing Translational Sciences UL1 TR000445 for Vanderbilt, and the Nutrition and Obesity Research Center at the University of Washington (P30 DK035816). 

**Table 1.** Baseline characteristics of study participants according to study group.

Clinical characteristics	All (n = 39)	Rilonacept (n = 19)	Placebo (n = 20)	p-value
Age, years (mean+SD)	65 ± 10	63 ± 12	68 ± 6	0.14
Race/Ethnicity, % (n)				0.87
White non-Hispanic	18 (46%)	8 (42%)	10 (50%)	
Hispanic	11 (28%)	6 (32%)	5 (25%)	
African American	10 (26%)	5 (25%)	5 (25%)	
Etiology of CKD, % (n)				
Hypertension	22 (56%)	12 (63%)	10 (50%)	0.41
Type II diabetes	18 (46%)	9 (47%)	9 (45%)	0.88
Type I diabetes	1 (3%)	0 (0%)	1 (5%)	0.32
ADPKD	3 (8%)	1 (5%)	2 (10%)	0.58
Renal vascular disease	3 (8%)	0 (0%)	3 (15%)	0.08
FSGS	1 (3%)	1 (5%)	0 (0%)	0.30
Antihypertensive agent, % (n)	39 (100%)	19 (100%)	20 (100%)	1.00
ACEi/ARB	26 (67%)	10 (53%)	16 (80%)	0.07
Diuretic	23 (59%)	12 (63%)	11 (55%)	0.52
Calcium channel blocker	20 (51%)	9 (47%)	11 (55%)	0.63
Beta blocker	16 (41%)	7 (37%)	9 (45%)	0.60
Statin, % (n)	22 (56%)	10 (53%)	12 (60%)	0.64
Smoking status, % (n)				0.05
Never	15 (39%)	8 (42%)	7 (35%)	
Current	4 (10%)	4 (21%)	0 (0%)	
Former	20 (51%)	7 (37%)	13 (65%)	
MDRD eGFR, mL/min/1.73m^2^ (mean ± SD)	38 ± 13	39 ± 14	38 ± 12	0.84
Urine protein/creatinine ratio, mg/mmol (median [interquartile range])	0.27 [0.11, 0.49]	0.34 [0.09, 0.65]	0.21 [0.11, 0.39]	0.61
BMI, kg/m^2^ (mean ± SD)	31.7 ± 5.2	32.6 ± 5.7	30.9 ± 4.6	0.30
SBP, mm Hg (mean ± SD)	133 ± 18	129 ± 15	137 ± 20	0.18
DBP, mm Hg (mean ± SD)	79 ± 11	79 ± 9	79 ± 12	0.95
Serum albumin, g/dL (mean ± SD)	3.9 ± 0.3	3.9 ± 0.3	3.9 ± 0.3	0.37
hsCRP, mg/L (median [interquartile range])	3.8 [1.6, 6.0]	4.7 [2.0, 8.5]	3.6 [1.3, 5.4]	0.09
Serum bicarbonate, mmol/L (mean ± SD)	24.2 ± 2.7	24.6 ± 2.8	25.5 ± 2.8	0.55

Data are n (%), mean ± SD, or median [interquartile range]. ADPKD = autosomal dominant polycystic kidney disease; CKD = chronic kidney disease; FSGS = focal segmental glomerular disease; ACEi = angiotensin converting enzyme inhibitor; ARB = angiotensin receptor blocker; MDRD = Modification of Diet in Renal Disease; eGFR = estimated glomerular filtration rate; BMI = body-mass index; SBP = systolic blood pressure; DBP = diastolic blood pressure. p-values are a comparison of rilonacept and placebo groups.

**Table 2.** Markers of chronic kidney disease-mineral and bone disorder according to study group.

Markers of CKD-MBD	Rilonacept (n = 19)		Placebo (n = 20)		p-value
	Baseline	12 weeks	Baseline	12 weeks	
Calcium (mg/dL)	9.3 ± 0.4	9.2 ± 0.6	9.5 ± 0.4	9.4 ± 0.4	0.28
Phosphorus (mg/dL)	3.6 ± 0.6	3.6 ± 0.8	3.6 ± 0.5	3.4 ± 0.6	0.30
25(OH)D (ng/mL)	28.2 ± 12.7	28.4 ± 12.3	33.5 ± 13.9	36.0 ± 13.0	0.28
1,25(OH)_2_D (pg/mL)	29.8 [25.3, 42.7]	35.8 [29.3, 46.7]	31.3 [24.1, 37.7]	34.9 [24.5, 47.0]	0.76
24,25(OH)_2_D_3_ (ng/mL)	1.04 [0.32, 2.31]	1.09 [0.52, 3.01]	3.43 [1.46, 4.26]	3.23 [1.85, 4.29]	0.78
iPTH (pg/mL)	75.7 [48.9, 157.4]	90.2 [52.8, 139.9]	82.4 [55.5, 117.6]	103.7 [82.3, 52.4]	0.98
FGF23 (pg/mL)	92.8 [61.5, 121.9]	73.8 [54.4, 162.5]	97.1 [70.3, 112.7]	70.6 [58.4, 108.4]	0.70

Data are mean ± SD or median [interquartile range]. CKD-MBD = chronic kidney disease-mineral and bone disorder; 25(OH)D = 25-hydroxyvitamin D; 1,25(OH)_2_D = 1,25-dihydroxyvitamin D; 24,25(OH)_2_D_3_ = 24,25-dihydroxyvitamin D_3_; iPTH = intact parathyroid hormone; FGF23 = fibroblast growth factor 23. p-values are for the comparison of change from baseline according to treatment arm using a two-sample t-test.

**Table 3. Table3:** Measures of physical and cognitive function according to study group.

Physical and cognitive function tests	Rilonacept (n = 12)		Placebo (n = 11)		p-value
	Baseline	12 weeks	Baseline	12 weeks	
400-m walk time (s)	312 ± 113	281 ± 72	275 ± 80	273 ± 77	0.07
TUG time (s)	9.0 ± 2.3	8.9 ± 1.8	8.8 ± 2.9	9.3 ± 5.0	0.48
Chair stands time (s)	14.3 ± 3.3	13.4 ± 2.8	15.3 ± 4.6	15.9 ± 6.8	0.35
Grip strength (dominant arm) (kg)	23.8 ± 8.3	25.3 ± 9.3	30.0 ± 12.6	29.7 ± 11.5	0.19
Rapid step time (s)	48.8 ± 16.7	60.0 ± 16.9	63.6 ± 50.1	63.0 ± 27.1	0.82
Rapid step errors (#)	7 ± 6	6 ± 6	4 ± 4	4 ± 4	0.58
Pegboard time (s)	110.9 ± 73.6	99.7 ± 58.2	89.7 ± 30.2	85.1 ± 27.7	0.35
Trail making A time (s)	44.2 ± 28.3	34.7 ± 14.4	44.8 ± 17.7	38.5 ± 22.0	0.66
Trail making A errors (#)	0.3 ± 0.7	0.1 ± 0.3	0.6 ± 0.7	0.2 ± 0.6	0.57
Trail making B time (s)	83.2 ± 28.2	64.4 ± 23.1	125.5 ± 81.6	128.3 ± 93.4	0.13
Trail making B errors (#)	0.9 ± 1.3	0.7 ± 1.3	1.3 ± 1.5	1.3 ± 1.3	0.79
Fatigue severity (score)	33.7 ± 11.7	32.8 ± 13.3	39.2 ± +12.4	39.0 ± 13.9	0.86

Data are mean ± SD. TUG = timed up-and-go test. p-values are for the comparison of change from baseline according to treatment arm using a two-sample t-test.

## Supplemental material

Supplemental Material. Supplementary Methods, Results, References, Tables, Figure.
