# Supplementary material for: Interleukin-1 inhibition, chronic kidney disease-mineral and bone disorder, and physical function
Source: Clin Nephrol. 2017 Jul 12;88(3):132–9. doi: 10.5414/CN109122 (PMC6595399; doi:10.5414/CN109122)
Supplement: Supplemental Material. [file clinnephrol-88-132-S01.pdf]

clinical nephrology
Online Supplement

©2017 Dustri-Verlag Dr. K. Feistle
ISSN 0301-0430

DOI 10.5414/CN109122
e-pub: July 12, 2017

Interleukin-1 inhibition, chronic kidney disease-mineral and bone disorder, and physical function

Kristen L. Nowak[1], Adriana Hung[2,3], Talat Alp Ikizler[2,3,4], Heather Farmer-Bailey[1], Natjalie Salas-Cruz[2], Sudipa Sarkar[2], Andrew Hoofnagle[5], Zhiying You[1], and Michel Chonchol[1]

[1]*University of Colorado Denver Anschutz Medical Campus, Aurora, CO,*
[2]*Vanderbilt University Medical Center,* [3]*VA Tennessee Valley Healthcare System,*
[4]*Vanderbilt Center for Kidney Disease, Nashville, TN, and*
[5]*University of Washington, Seattle, WA, USA*

Supplemental material

Supplementary methods

Study design

The details of the parent study have been published previously [1]. Briefly, a 12-week, randomized, placebo-controlled (1 : 1 allocation), parallel group, double-blind study was conducted at two sites (the University of Colorado Denver Anschutz Medical Campus and the Tennessee Valley Healthcare System/Vanderbilt University Medical Center) between September 2012 and September 2014. The co-primary outcomes were change in brachial artery flow-mediated dilation and aortic pulse-wave velocity in the rilonacept compared to placebo group, and these results have been published previously. Secondary outcomes, determined a priori, that were evaluated in the present analysis were changes in serum markers of chronic kidney disease-mineral and bone disorder (CKD-MBD) and physical/cognitive function.

Study participants

Patients eligible for inclusion were men and women 18 – 80 years of age with stage 3 – 4 CKD (estimated glomerular filtration rate [eGFR] with the 4-variable Modified Diet Renal Disease [MDRD] prediction equation [2] 15 – 60 mL/min/1.73m^2 and stable renal function in the past 3 months) and evidence of chronic inflammation (hsCRP > 2.0 mg/L and < 30 mg/L on at least 2 consecutive weekly determinations (to identify chronic inflammation rather than acute inflammation/infection)). Detailed inclusion and exclusion criteria have been published previously [1]. All participants who completed the parent study were included in the analysis of markers of CKD-MBD (n = 19 rilonacept and n = 20 placebo). A subgroup of participants from the Denver site additionally completed a battery of tests to evaluate various domains of cognitive and physical function (n = 12 rilonacept and n = 11 placebo). All procedures were approved by the Institutional Review Board of the University of Colorado Denver, the Tennessee Valley Healthcare System, and Vanderbilt University Medical Center and adhere to the Declaration of Helsinki. The nature, benefits, and risks of the study were explained to the volunteers and their written informed consent was obtained prior to participation. The trial was registered at ClinicalTrials.gov (NCT01663103).

Procedures

Rilonacept and weekly visits

These details have been published previously [1]. Briefly, rilonacept, a soluble IL-1 decoy receptor, or placebo was injected subcutaneously (320-mg loading dose followed by 160 mg/week for 12 weeks). Vital signs and a careful evaluation of symptoms and signs of upper respiratory infection were performed weekly prior to delivery of the injection.

Received
January 19, 2017;
accepted in revised form
May 5, 2017

Correspondence to
Kristen L. Nowak, PhD, MPH
Division of Renal Diseases and Hypertension, University of Colorado Denver,
12700 E 19th Ave C281, Aurora, CO 80220, USA
Kristen.Nowak@ucdenver.edu

Markers of CKD-MBD

Serum samples were stored at –80 °C until time of analysis at the University of Washington (2015). The stability of vitamin D metabolites and fibroblast growth factor 23 (FGF23) in frozen samples has been well described previously [3]. Total serum 25-hydroxyvitamin D (25(OH)D; sum of 25(OH) D_2 and 25(OH)D_3), 1,25-dihydroxyvitamin D (1,25$(OH)_2$D [sum of 1,25$(OH)_2D_2$ and 1,25$(OH)_2D_3$]), and 24,25-dihydroxyvitamin D (24,25$(OH)_2D_3$; the most abundant product of vitamin D catabolism and reflection of vitamin D activity [4]), were measured using immunoaffinity purification and liquid chromatography-tandem mass spectrometry [5]. Calibration was confirmed with the National Institute of Standards and Technology's standard reference material. Intact FGF23 was measured using the Kainos immunoassay, which has been shown to be the most sensitive assay for FGF23 [6] and detects the full-length, biologically-intact FGF23 molecule via midmolecule and distal epitopes [5]. The lower limits of quantification for 25(OH)D_2 and 25(OH)D_3 were 1 ng/mL and 1 ng/mL, with a between-assay imprecision of 5.99 – 6.72%CV at 9.3 – 26.2 ng/mL and 3.54 – 4.41%CV at 9.5 – 32.3 ng/mL, respectively. Limits of quantification for 1,25$(OH)_2D_2$ and 1,25$(OH)_2D_3$ were 5 pg/mL and 5 pg/mL, with a between-assay imprecision of 8.92 – 9.91%CV at 14.2 – 33.8 pg/mL and 7.95 – 10.40%CV at 18.1 – 47.8 pg/mL, respectively. The lower limit of detection of 24,25$(OH)_2D_3$ was 0.5 ng/mL, and the between-assay imprecision was 5.17 – 7.42%CV at 1.3 – 4.6 ng/mL. The interassay CV for FGF23 was 6.3 – 8.3% 43 – 894 pg/mL. Intact parathyroid hormone (iPTH) was measured using a 2-site immunoassay on a Beckman Dxl automated clinical analyzer. The reference range, determined from the central 95% of values from 43 laboratory personnel controls with normal 25(OH)D concentrations, was 17 – 65 pg/mL. Serum calcium and phosphorus were measured using standard techniques. All interassay CVs were determined using two blinded quality-control specimens placed in random positions in each batch.

Physical and cognitive function

These measurements were performed at the Health and Wellness Center at the University of Colorado Anschutz Medical Campus. Endurance was assessed as time to briskly walk 400 m, performed as ~ 3 laps around an indoor track [7]. Mobility was quantified using a timed up and go test as the time to stand unassisted, walk to a target 3 m from the chair, and return to a seated position (mean of 3 trials) [7]. Muscle strength was evaluated using the five-repetition sit-to-stand test, evaluating time to stand up and sit down from a chair as quickly as possible with arms folded across the chest (mean of 3 trials) [8]. Grip strength was evaluated as the maximal voluntary isometric contraction of the dominant arm for ~ 3 seconds using a standard dynamometer (hydraulic hand dynamometer, 200 lb capacity, Lafayette Instruments (Lafayette, IN, USA); mean of 3 – 5 trials) [7]. Balance was evaluated as the time and number of errors during a rapid step test, as described previously [7]. Dexterity was measured as time to complete the grooved pegboard test (25-hole pegboard, Lafayette Instruments; mean of 3 trials) [7]. The trail making tests A and B were administered as indices of cognitive function [9]. Self-reported perception of fatigue in daily life was assessed using the Fatigue Severity Scale [10].

Statistics

Differences in baseline variables between treatment groups were assessed using t-tests, rank-based tests, or χ^2-tests. The changes in markers of CKD-MBD and physical/cognitive function in response to treatment were analyzed by comparing change from baseline for each outcome between study groups using a two-sample t-test. Skewed variables were log-transformed prior to analyses. No adjustment was made for multiple comparisons as all outcomes in this secondary analysis were considered exploratory. The evaluation of physical and cognitive function was performed as a pilot study in a subgroup of participants. Due to the small sample size, analyses were not stratified by sex. All data are reported as mean ± SD or medians (interquartile range). SAS software (version 9.4) was used for all analyses.

Supplementary results

Table 1, Table 2, and Figure 1.

References

[1] *Nowak KL, Chonchol M, Ikizler TA, Farmer-Bailey H, Salas N, Chaudhury R, Wang W, Smits G, Tengesdal I, Dinarello CA, Hung A*. IL-1 inhibition and vascular function in CKD. J Am Soc Nephrol. 2017; *28:* 971-980. PubMed

[2] *Levey AS, Bosch JP, Lewis JB, Greene T, Rogers N, Roth D*. A more accurate method to estimate glomerular filtration rate from serum creatinine: a new prediction equation. Modification of Diet in Renal Disease Study Group. Ann Intern Med. 1999; *130:* 461-470. PubMed

[3] *Kendrick J, Cheung AK, Kaufman JS, Greene T, Roberts WL, Smits G, Chonchol M*; *HOST Investigators*. FGF-23 associates with death, cardiovascular events, and initiation of chronic dialysis. J Am Soc Nephrol. 2011; *22:* 1913-1922. CrossRef PubMed

[4] *Bosworth CR, Levin G, Robinson-Cohen C, Hoofnagle AN, Ruzinski J, Young B, Schwartz SM, Himmelfarb J, Kestenbaum B, de Boer IH*. The serum 24,25-dihydroxyvitamin D concentration, a marker of vitamin D catabolism, is reduced in chronic kidney disease. Kidney Int. 2012; *82:* 693-700. CrossRef PubMed

[5] *Chonchol M, Greene T, Zhang Y, Hoofnagle AN, Cheung AK*. Low vitamin D and high fibroblast growth factor 23 serum levels associate with infectious and cardiac deaths in the HEMO Study. J Am Soc Nephrol. 2016; *27:* 227-237. CrossRef PubMed

[6] *Imel EA, Peacock M, Pitukcheewanont P, Heller HJ, Ward LM, Shulman D, Kassem M, Rackoff P, Zimering M, Dalkin A, Drobny E, Colussi G, Shaker JL, Hoogendoorn EH, Hui SL, Econs MJ*. Sensitivity of fibroblast growth factor 23 measurements in tumor-induced osteomalacia. J Clin Endocrinol Metab. 2006; *91:* 2055-2061. CrossRef PubMed

Supplemental Table 1. Baseline characteristics of participants in the substudy according to study group.

Clinical characteristics	All (n = 21)	Rilonacept (n = 12)	Placebo (n = 11)	p-value
Age, years (mean ± SD)	65 ± 10	63 ± 12	67 ± 7	0.33
Race/Ethnicity, % (n)				0.78
White non-Hispanic	11 (48%)	6 (50%)	5 (46%)	
Hispanic	9 (39%)	5 (42%)	4 (36%)	
African American	3 (13%)	1 (8%)	2 (18%)	
Etiology of CKD, % (n)				
Hypertension	14 (61%)	9 (75%)	5 (46%)	0.15
Type II diabetes	8 (35%)	5 (42%)	3 (27%)	0.47
Type I diabetes	1 (4%)	0 (0%)	1 (9%)	0.29
ADPKD	3 (13%)	1 (8%)	2 (18%)	0.48
Renal vascular disease	3 (13%)	0 (0%)	3 (27%)	0.05
FSGS	0 (0%)	0 (0%)	0 (0%)	1.00
Antihypertensive agent, % (n)	21 (100%)	12 (100%)	11 (100%)	1.00
ACEi/ARB	15 (65%)	5 (42%)	10 (91%)	0.01
Diuretic	14 (61%)	8 (67%)	6 (55%)	0.55
Calcium channel blocker	13 (57%)	6 (50%)	7 (64%)	0.51
Beta blocker	9 (39%)	4 (33%)	5 (46%)	0.55
Statin, % (n)	11 (48%)	5 (42%)	6 (55%)	0.54
Smoking status, % (n)				0.30
Never	9 (39%)	5 (42%)	4 (46%)	
Current	2 (9%)	2 (17%)	0 (0%)	
Former	12 (52%)	5 (42%)	7 (63%)	
MDRD eGFR, mL/min/1.73m^2 (mean ± SD)	33 ± 65	32 ± 9	34 ± 12	0.76
Urine protein/creatinine ratio, mg/mmol (median [interquartile range])	0.23 [0.09, 0.47]	0.36 [0.18, 0.57]	0.20 [0.07, 0.23]	0.14
BMI, kg/m^2 (mean ± SD)	30.1 ± 4.3	30.1 ± 4.5	29.3 ± 4.0	0.38
SBP, mm Hg (mean ± SD)	129 ± 14	128 ± 14	130 ± 14	0.75
DBP, mm Hg (mean ± SD)	79 ± 12	79 ± 11	78 ± 13	0.94
Serum albumin (g/dL)	3.8 ± 0.2	3.7 ± 0.2	3.8 ± 0.2	0.29
hsCRP, mg/L (median [interquartile range])	7.2 [1.9, 9.2]	1.4 [1.0, 3.8]	3.1 [1.0, 7.8]	0.02
Serum bicarbonate, mmol/L (mean ± SD)	23.2 ± 2.4	23.2 ± 2.6	23.3 ± 2.2	0.92

Data are n (%), mean ± SD, or median [interquartile range]. CKD = chronic kidney disease; ADPKD = autosomal dominant polycystic kidney disease; FSGS = focal segmental glomerular disease; ACEi = angiotensin-converting enzyme inhibitor; ARB = angiotensin receptor blocker; MDRD = Modification of Diet in Renal Disease; eGFR = estimated glomerular filtration rate; BMI = body-mass index; SBP = systolic blood pressure; DBP = diastolic blood pressure; hsCRP = high-sensitivity C-reactive protein. p-values are a comparison of rilonacept and placebo groups.

[7] *Justice JN, Johnson LC, DeVan AE, Cruickshank-Quinn C, Reisdorph N, Bassett CJ, Evans TD, Brooks FA, Bryan NS, Chonchol MB, Giordano T, McQueen MB, Seals DR*. Improved motor and cognitive performance with sodium nitrite supplementation is related to small metabolite signatures: a pilot trial in middle-aged and older adults. Aging (Albany NY). 2015; *7:* 1004-1021. CrossRef PubMed

[8] *Visser M, Kritchevsky SB, Goodpaster BH, Newman AB, Nevitt M, Stamm E, Harris TB*. Leg muscle mass and composition in relation to lower extremity performance in men and women aged 70 to 79: the health, aging and body composition study. J Am Geriatr Soc. 2002; *50:* 897-904. CrossRef PubMed

[9] *Kurella M, Chertow GM, Luan J, Yaffe K*. Cognitive impairment in chronic kidney disease. J Am Geriatr Soc. 2004; *52:* 1863-1869. CrossRef PubMed

[10] *Bonner A, Wellard S, Caltabiano M*. The impact of fatigue on daily activity in people with chronic kidney disease. J Clin Nurs. 2010; *19:* 3006-3015. CrossRef PubMed

Supplemental Table 2. Baseline values for markers of chronic kidney disease-mineral and bone disorder and measures of physical and cognitive function according to study group.

Markers of CKD-MBD	All (n = 39)	Rilonacept (n = 19)	Placebo (n = 20)	p-value
Calcium (mg/dL)	9.4 ± 0.4	9.3 ± 0.4	9.5 ± 0.4	0.06
Phosphorus (mg/dL)	3.6 ± 0.6	3.6 ± 0.6	3.6 ± 0.5	0.74
25(OH)D (ng/mL)	31.0 ± 13.4	28.2 ± 12.7	33.5 ± 13.9	0.18
$1,25(OH)_2D$ (pg/mL)	30.2 [24.4, 42.7]	29.8 [25.3, 42.7]	31.3 [24.1, 37.7]	0.59
$24,25(OH)_2D_3$ (ng/mL)	2.07 [0.93, 3.86]	1.04 [0.32, 2.31]	3.43 [1.46, 4.26]	0.002
iPTH (pg/mL)	81.7 [55.5, 117.6]	75.7 [48.9, 157.4]	82.4 [55.5, 117.6]	0.62
FGF23 (pg/mL)	93.2 [70.1, 115.5]	92.8 [61.5, 121.9]	97.1 [70.3, 112.7]	0.94
Physical and cognitive function tests	All (n = 21)	Rilonacept (n = 12)	Placebo (n = 11)	p-value
400-m walk time (s)	291 ± 95	312 ± 113	275 ± 80	0.42
TUG time (s)	8.0 ± 2.6	9.0 ± 2.3	8.8 ± 2.9	0.86
Chair stands time (s)	14.8 ± 3.9	14.3 ± 3.3	15.3 ± 4.6	0.57
Grip strength (dominant arm) (kg)	26.8 ± 10.8	23.8 ± 8.3	30.0 ± 12.6	0.18
Rapid step time (s)	61.4 ± 15.4	48.8 ± 16.7	63.6 ± 50.1	0.50
Rapid step errors (#)	6 ± 5	7 ± 6	4 ± 4	0.25
Pegboard time (s)	100.3 ± 55.9	110.9 ± 73.6	89.7 ± 30.2	0.39
Trail making A time (s)	44.5 ± 23.3	44.2 ± 28.3	44.8 ± 17.7	0.96
Trail making A errors (#)	0.4 ± 0.7	0.3 ± 0.7	0.6 ± 0.7	0.28
Trail making B time (s)	104.4 ± 63.3	83.2 ± 28.2	125.5 ± 81.6	0.14
Trail making B errors (#)	1.1 ± 1.4	0.9 ± 1.3	1.3 ± 1.5	0.53
Fatigue severity (score)	36.3 ± 12.1	33.7 ± 11.7	39.2 ± 12.4	0.28

Data are mean ± SD or median [interquartile range]. CKD-MBD = chronic kidney disease-mineral and bone disorder; 25(OH)D = 25-hydroxyvitamin D; $1,25(OH)_2D$ = 1,25-dihydroxyvitamin D; $24,25(OH)_2D_3$ = 24,25-dihydroxyvitamin D_3; iPTH = intact parathyroid hormone; FGF23 = fibroblast growth factor 23; TUG = timed up-and-go test. p-values are a comparison of rilonacept and placebo groups.

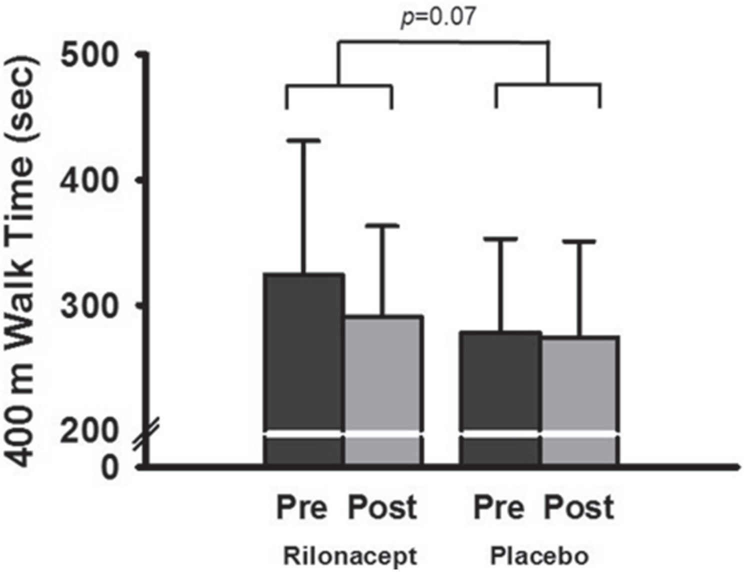

Supplemental Figure 1. Effect of interleukin-1 inhibition on endurance. 400 m walk time, an index of endurance, at baseline (black bars) and following 12 weeks of treatment (gray bars) with either rilonacept or placebo. Values are mean ± SD.
